# Children’s and parents’ perceptions concerning surgical attire: a systematic review

**DOI:** 10.1590/1984-0462/2022/40/2020380

**Published:** 2021-10-25

**Authors:** Luciana Butini Oliveira, Carla Massignan, Isabel Cristina Quaresma Rêgo, Maria Marlene de Souza Pires, Bruce Dick, Michele Bolan, Graziela De Luca Canto

**Affiliations:** a Faculdade São Leopoldo Mandic, Campinas, SP, Brazil.; b Universidade de Brasília, Brasília, DF, Brazil.; c Universidade de Santa Catarina, Florianópolis, SC, Brazil.; d University of Alberta, Edmonton, Canada.

**Keywords:** Child, Parents, Perception, Physicians, Criança, Pais, Percepção, Médicos

## Abstract

**Objective:**

To review the literature about children’s and parent’s perceptions on surgical attire.

**Data source:**

A systematic search was conducted in the databases EMBASE, Latin American and Caribbean Health Sciences (LILACS), PubMed, PsycINFO, Scopus and Web of Science. Grey literature was searched on Google Scholar, Open Grey and ProQuest Dissertations, and Theses Database.

**Data synthesis:**

A total of 2,567 papers were identified. After a two-phase selection, 15 studies were included in narrative synthesis. Children favored wearing white coats in five of the nine included studies (55.5% [95%CI 48.3-62.7]; p=1.00). With respect to parents’ preferences, results of vote counting showed that in 11 of 15 included studies, they favored physicians wearing white coats (73.3% [95%CI 67.9-78.6]; p=0.11).

**Conclusions:**

Children and parents have preferred physicians to wear a white coat with a very low certainty of evidence.

## RESUMO

**Objetivo:**

Revisar a literatura sobre as percepções de crianças e seus pais a respeito de trajes médicos.

**Fontes de dados:**

Buscas sistemáticas foram conduzidas nas bases de dados EMBASE, Literatura Latino-americana e do Caribe em Ciências da Saúde (LILACS), PubMed, PsycINFO, Scopus e Web of Science. A literatura cinzenta foi pesquisada no Google Scholar, Open Grey e ProQuest Dissertations e Theses Database.

**Síntese dos dados:**

Foram identificadas 2.567 publicações e, após uma seleção de duas fases, foram incluídos 15 estudos na síntese qualitativa. As crianças tinham preferência pelo uso de jaleco branco em cinco dos nove estudos incluídos (55,5% [IC95% 48,3-62,7]; p=1,00). Em 11 dos 15 estudos incluídos, os pais tinham preferência pelos médicos que utilizavam jaleco (73,3% [IC95% 67,9-78,6]; p=0,11).

**Conclusões:**

Com uma qualidade baixa de evidência, crianças e pais preferem os médicos que usam jaleco branco.

## INTRODUCTION

Attire plays an important role in many professions. In pediatric populations, the pediatrician’s appearance has been considered a crucial element that may affect the confidence and the comfort of both children and parents. Some studies in the literature have investigated the impact of physicians’ attire on the reliance and confidence of patients.^1^ ^,^ ^2^ ^,^ ^3^ ^,^ ^4^ Previous studies have also evaluated the perception/preferences of parents and their children, and the results have been conflicting.^5^ ^,^ ^6^

Physicians’ attire can be considered as professionalism indicator, which could impact the patient-doctor relationship.^7^ However, children can perceive surgical attire differently from their parents.^5^ In fact, the pediatricians’ wearing white coats during children’s care is considered a dilemma, often debated, due to the fact that a white coat can be intimidating for children.^8^ ^,^ ^9^ A previous study has shown that most children do not find face shields or surgical masks frightening, however, they prefer physicians in clear plastic face shields so that they can see the physicians’ faces. Parents have poorly predicted what their children would prefer in studies that have explored the use of face shields *versus* masks.^10^ In addition to patients’ preferences, considering the risk of bacterial contamination and the risk of infection transmission when evaluating attire choices is important. Wearing white coats by physicians has generally been accepted and adopted in daily routine. However, more recently, it has been recognized that surgical attire may play an essential role in transmitting infections within and outside hospital settings.^6^

The COVID-19 pandemic has changed the personal protective equipment (PPI) required for routine medical care. Currently, face shield, mask, gowns, and eye protection are often among the precautionary equipment that clinicians are required to wear. A study regarding severe acute respiratory syndrome (SARS) found that 17.5% of 174 children and 0.0% of their parents appreciated professionals wearing protective equipment compared to physicians dressed in formal attire, such as a white coat.^11^ To date, there is no study that has investigated parents’ and children’s perceptions about the COVID-19 different attires *versus* standard personal protective equipment.

A previous systematic review^12^ has examined the influence of physician attire on patient’s perceptions, including trust/reliance, satisfaction, and confidence. However, in such research, studies involving pediatric patients were excluded. Thus, this systematic review has aimed at answering the question: “What are children’s and parents’ perceptions regarding physicians’ attire?

## METHOD

This review was registered in The Open Science Framework (OSF) under DOI 10.17605/OSF.IO/MK8U9.^13^ This systematic review followed the Preferred Reporting Items for Systematic Review and Meta-Analysis (PRISMA) checklist.^14^ The Synthesis without meta-analysis (SWiM) in systematic reviews reporting guideline was also adopted.^15^

This systematic review has been guided by the focused question: “What are children’s and parents’ perceptions of physicians’ attire?” To be included, descriptive studies were meant to evaluate children’s preferences (or perceptions) concerning physicians’ attire. Any kind of method used to assess children’s preference or perception as to physician’s attire (e.g.: questionnaire, images) were included. Studies with different objectives have been excluded. Secondary studies (articles review, letter to the editor, books, book chapters, etc.) and those with adult population were also excluded.

An experienced health sciences librarian helped with the search strategy and with appropriate modification for each database. The databases EMBASE, Latin American and Caribbean Health Sciences (LILACS), PubMed, PsycINFO, Scopus and Web of Science were searched from their inception to June 1^st^, 2019 and updated on May 28^th^, 2020. Grey literature was searched on Google Scholar, limited to the first 100 most relevant articles, the database System for Information on Grey Literature in Europe (OpenGrey). ProQuest Dissertations and Theses Database were also searched.

The reference lists of the studies included were also investigated to identify additional studies. EndNote ® X7 (Thomson Reuters, New York, USA) and Rayyan software^16^ (http://rayyan.qcri.org/) were used to manage references and to identify and remove duplicate hits.

Two independent reviewers (CM, LB) performed the selection process in two phases. Firstly, they assessed all retrieved titles and abstracts for eligibility. Secondly, the full-text articles were obtained and evaluated in cases in which both reviewers considered the abstracts to be potentially relevant. Disagreements were settled by discussion involving a third reviewer (MB).

For data extraction, two reviewers (CM, LB) independently collected data in pre-piloted forms; their findings were compared. Any disagreement was solved by mutual agreement between the authors.^17^ The following data were extracted from the included studies: authors, year, country, sample characteristics (sample size, sex, age), objectives, study characteristics (setting), and outcome characteristics (data analysis, findings, direction of the effect, and main conclusion).

Two reviewers (CM, LB) have independently assessed the methodological quality of studies included by using the checklist from the Joanna Briggs Institute.^18^ The questionnaire for Analytical Cross-Sectional Studies was applied, and all domains in the questionnaire were considered.

Data based on vote counting was summarized, taking into consideration the direction of the effect.^14^ ^,^ ^17^ The primary outcome was proportion of parents and children favorable to physicians wearing white coats. Each study included was categorized according to “in favor of the physician wearing a white coat” or “not in favor of the physician wearing a white coat”. The probability of observing preference favoring a white gown for parents and for children was calculated using a binomial probability test with a 95% confidence interval (95%CI). Parents and children were considered separately. The sign test was used to compare the number of studies with parents and children that favored the use of a white coat with the number of studies with parents and children that did not favor the use of a white coat irrespective of whether the findings were statically significant, as suggested by Borenstein et al.^18^ In the test, one expects half of the studies to be positioned on each side of the non-effect line. Therefore, the number of studies, the number of effects favoring white coat and the null value of 0.5 were entered in an Excel spreadsheet. The results are presented in a table organized based on the characteristics of the studies’ populations (i.e., country and if studies addressed parents, children, or both). A harvest plot was also built to visually compare the results.^19^ The plot also presents the quality assessment, with taller bars representing low risk of bias and shorter bars indicating moderate risk of bias. A meta-analysis was not performed due to clinical (clinics, hospitals, cultural differences among populations) and methodological (picture based, questionnaire based) heterogeneity across the studies included.

Two reviewers (LBO and CM) independently analyzed the certainty of evidence using the Grading of Recommendations, Assessment, Development and Evaluation (GRADE) criteria.^20^ In observational studies, this system starts with a low grade and can be either upgraded or downgraded. Aspects such as risk of bias, inconsistency, indirectness, imprecision, and publication bias are reasons to lower the certainty rating of evidence and the presence of a large effect. Dose response gradient and controlling of plausible confounders are causes of increasing this rating in observational studies. Of note, due to the nature of the present study analysis, assessing the consistency of effects was not possible.^15^

Additional materials on search strategies used in databases, excluded articles and reasons for exclusion, and detailed bias risk assessment information are available with the corresponding author.

## RESULTS

**Figure 1:**
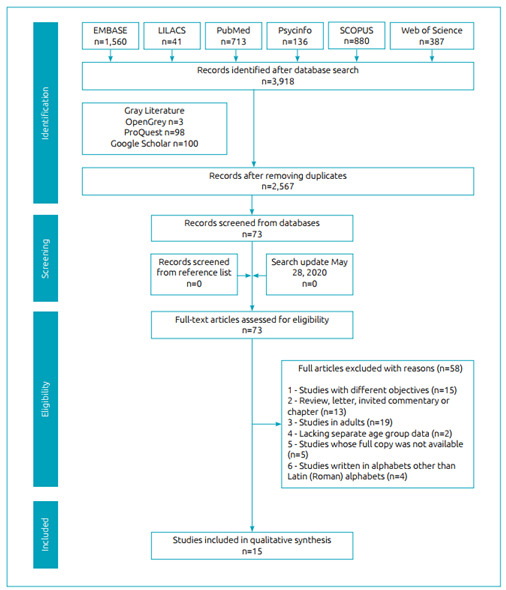
Flow diagram of literature search and selection criteria.

During the initial search (Phase 1), 2,567 different studies were identified across the six electronic databases after duplicates were removed. Following a comprehensive evaluation of the abstracts, 73 articles were deemed potentially useful, and were selected for Phase 2 assessment. There were no additional citations identified from the grey literature search. From these 73 remaining studies, 58 were subsequently excluded. Thus, 15 studies^5^ ^,^ ^6^ ^,^ ^11^ ^,^ ^21^ ^,^ ^22^ ^,^ ^23^ ^,^ ^24^ ^,^ ^25^ ^,^ ^26^ ^,^ ^27^ ^,^ ^28^ ^,^ ^29^ ^,^ ^30^ ^,^ ^31^ ^,^ ^32^ were included in narrative analysis. No additional study that might have been inadvertently missed by the search procedures was identified after further reviewing the reference lists of the 15 included studies. A flow chart of the process of identification, inclusion, and exclusion of studies is shown in Figure 1.

All the studies included used a descriptive design. The geographical location of the research teams who published the included studies were as follows: two from Saudi Arabia,^5^ ^,^ ^6^ two from India,^31^ ^,^ ^32^ seven from the USA,^22^ ^,^ ^24^ ^,^ ^25^ ^,^ ^27^ ^,^ ^28^ ^,^ ^29^ ^,^ ^30^ two from Canada,^11^ ^,^ ^33^ one from Austria,^23^ and one from France.^26^

Sample size ranged widely, from 40^33^ to 450 subjects.^30^ Six studies adopted a picture-based survey and questionnaire.^5^ ^,^ ^6^ ^,^ ^22^ ^,^ ^23^ ^,^ ^25^ ^,^ ^27^. Five studies adopted a questionnaire based-survey, ^24^ ^,^ ^29^ ^,^ ^30^ ^,^ ^31^ ^,^ ^32^ and four conducted a picture-based survey.^11^ ^,^ ^26^ ^,^ ^28^ ^,^ ^33^

Most studies included were carried out in hospitals,^6^ ^,^ ^22^ ^,^ ^23^ ^,^ ^25^ ^,^ ^26^ ^,^ ^27^ ^,^ ^28^ ^,^ ^31^ ^,^ ^32^ ^,^ ^33^ whereas others took place in clinics,^11^ ^,^ ^24^ ^,^ ^29^ as well as one located in a University setting.^5^

Nine studies included samples of both parents and children,^5^ ^,^ ^11^ ^,^ ^23^ ^,^ ^24^ ^,^ ^25^ ^,^ ^26^ ^,^ ^27^ ^,^ ^28^ ^,^ ^29^ and six included samples of parents only.^6^ ^,^ ^22^ ^,^ ^30^ ^,^ ^31^ ^,^ ^32^ ^,^ ^33^ Of the 15 included studies, the results of 11 studies found that parents prefer their doctors to wear white coat attire.^5^ ^,^ ^6^ ^,^ ^11^ ^,^ ^22^ ^,^ ^25^ ^,^ ^26^ ^,^ ^27^ ^,^ ^29^ ^,^ ^31^ ^,^ ^32^ ^,^ ^33^ Five studies suggested that children preferred their doctors to wear white coat attire.^26^ ^,^ ^27^ ^,^ ^28^ ^,^ ^29^ ^,^ ^33^ In addition, four studies that addressed both children’s and parent’s perceptions on physicians’ attire demonstrated that parents and children both preferred physicians to wear a white coat.^26^ ^,^ ^27^ ^,^ ^29^ ^,^ ^33^ A summary of the study’s descriptive characteristics and the main results from the 15 studies included can be found in Table 1.

**Table 1:** Summary of descriptive characteristics of articles included from Asian countries that evaluated perception of pediatric physicians’ attire by parents, children, and adolescents (n=4).

Author(s), year, country, setting	Study sample (n), sex, and age (years old)	Objectives	Overall Results Favors white coat (+) Does not favor white coat (-)
a) Alnasser et al.,^5^ 2016, Saudi Arábia, University	**Parents and children** Parents 99; females 91, <20 >40 y.o. Children 33, 11 females, 22 males, 6-12 y.o.	To assess perceptions of Saudi children and parents toward physicians’ attire within inpatient general pediatrics settings.	Parents (+) Children (-)
b) Aldrees et al.,^6^ 2017, Saudi Arabia, Hospital	**Parents only** 259; all females, 32 y.o. or younger	To assess Saudi mother’s preferences regarding Saudi children’s physicians’ attire, and its influence on parents’ level of trust and confidence.	Parents (+)
c) Raichur et al.,^31^ 2001, India, Hospital	**Parents only** 210; sex and age not informed.	To assess parents’ opinions regarding attire and appearance of pediatricians.	Parents (+)
d) Solanki et al.,^32^ 2015, India, Hospital	**Parents only** 400; sex and age - not informed.	To study the ideas of parents about dressing and attire of the pediatrician.	Parents (+)

**Figure 2:**
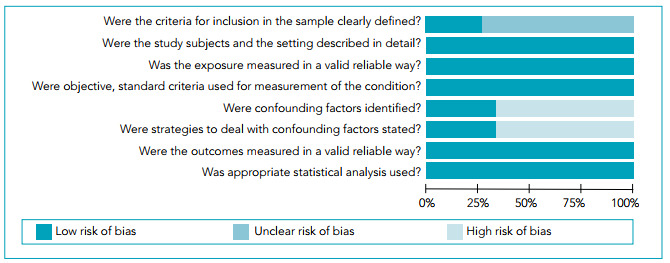
Risk of bias graph: Review authors’ judgements as to each risk of bias item presented as percentages across all studies included.

Most studies included (n=8) were evaluated to have low risk of bias; the remaining seven were found to have moderate risk. Although the studies were all constructed using the same study design, the primary identified methodological issue concerned the study samples. Most studies included used a convenience sample, which is at high risk of not being truly representative of the general population. The main flaws that studies presented were related to a lack of clearly reported criteria for inclusion in the sample, problems with identifying confounding factors, and reporting whether strategies to deal with confounding factors were adopted. More information about the risk of bias of included studies can be found in Figure 2.

**Table 2:** Summary of descriptive characteristics of articles included from the United States of America that evaluated perception of pediatric physicians’ attire by parents, children, and adolescents (n=7).

Author(s), year, country, setting	Study sample (n), sex, and age (years old)	Objectives	Overall Results Favors white coat (+) Does not favor white coat (-)
e) Longmuir et al.,^24^ 2010, United States, Clinic	**Parents and children** Total 227, sex and age - not informed	To determine if patients and their families have a preference regarding physician and staff attire.	Parents (-) Children (-)
f) Marino et al.,^25^ 1991, United States, Hospital	**Parents and children** Parents 50; 84% female; 25-35 y.o. Children 50; 58% female; 5-8 y.o.	To evaluate perceptions of a pediatrician’s attire.	Parents (+) Children (-)
g) Matsui et al.,^27^ 1998, United States, Hospital	**Parents and children** Parents 100; 82.3% female; 24-46 y.o. Children 100; sex not - not informed; 4-8 y.o.	To determine if young children have a preference regarding whether physicians do or do not wear a white coat.	Parents (+) Children (+)
h) McCarthy et al.,^28^ 1999, United States, Hospital	**Parents and children** Parents 50 sex and age not informed. Children 50; 25 female; 5-15 y.o.	To evaluate the child’s and parents’ visual perception of physicians.	Parents (-) Children (+)
i) Muran and Gold,^29^ 1990, United States, Clinics	**Parents and children** Parents 284; sex and age - not informed. Children 159 sex - not informed; 1-18 y.o.	To evaluate patients’ and parents’ expectations regarding physician attire.	Parents (+) Children (+)
j) Gonzalez Del Rey and Paul 1995,^22^ United States, Hospital	**Parents only** 360; 252 females, 68% between 19 and 40 y.o.	To determine parent preference for pediatric emergency physicians’ attire and to investigate if variables, including severity of illness, sex, race, age, insurance status, time, and type of emergency department visit influence preferences.	Parents (+)
k) Nibhanipudi et al.,^30^ 2013, United States, not informed	**Parents only** 450; sex and age - not informed.	To determine Spanish-speaking parents’ acceptance of the physician’s attire in the pediatric emergency department.	Parents (-)

**Table 3:** Summary of descriptive characteristics of articles included from Canada that evaluated perception of pediatric physicians’ attire by parents and children (n=2).

Author(s), year, country, setting	Study sample (n), sex, and age (years old)	Objectives	Overall Results Favors white coat (+) Does not favor white coat (-)
l) Truong et al.,^11^ 2006, Canada, Tertiary care center	**Parents and children** Parents 174; 72.4% female; 18-60 y.o. Children 197, sex not reported; 4-8 y.o.	To determine if young children have a preference regarding whether physicians wear standard precautions attire.	Parents (+) Children (+)
m) Taylor,^33^ 1987, Canada, Hospital	**Parents only** 40; female 72%; 17-51 y.o.	To test the hypothesis that parents have preference regarding physician’s attire	Parents (+)

**Table 4:** Summary of descriptive characteristics of articles included from European countries that evaluated perception of pediatric physicians’ attire by parents, children, and adolescents (n=2).

Author(s), year, country, setting	Study sample (n), sex, and age (years old)	Objectives	Overall Results Favors white coat (+) Does not favor white coat (-)
n) Hofmann et al,.^23^ 2012, Austria, Hospital	**Parents and children** Parents 72; sex and age not reported Children 55; 6-18 y.o. Children 40; 0-6 y.o.	To find out whether the different attire of a pediatrician have an influence on the children’s and parents’ opinion about the doctor.	Parents (-) Children (-)
o) Maruani et al.,^26^ 2013, France, Hospital	**Parents and children** Hospital: Parents, 50; 46 female; 39.0±5.8 Children 50; 20 female, 7-11 y.o. Teenagers 50; 29 female; 11-17 y.o. Private-practice: Parents, 24; 20 female; 39.0±6.2 Children 27; 16 female, 7-11 y.o. Teenagers 30; 15 female; 11-17 y.o.	To determine whether dressing style (professional white coat or formal, semiformal, or casual attire) affects patients’ confidence (children, teenagers, adults) in the physician with dermatology complaints consulting in the hospital or private practice.	Parents (+) Children (+)

**Figure 3:**
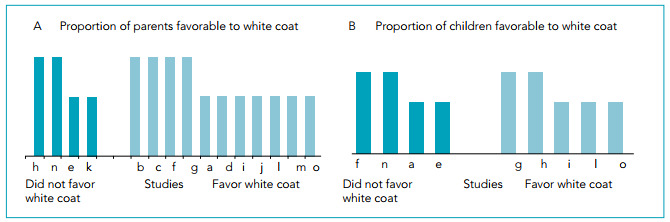
Harvest plots representing proportions of parents (A) and children (B) that favor white coat. Columns represent individual studies with indication of references. Height depicts overall quality assessment judgment (tall=low risk of bias; short=moderate risk of bias).

The available effect estimates are presented in Tables 1, 2, 3, and 4 (column 4). With respect to parents’ preferences, results of vote counting showed that in 11 of the 15 studies included, they favored physicians wearing white coats (73.3% (95%CI 67.9-78.6); p=0.11) (Figure 3A). Children favored wearing white coats in five out of the nine studies included (55.5% (95%CI 48.3-62.7); p=1.00) (Figure 3B).

The confidence in cumulative evidence, defined using GRADE criteria,^20^ was evaluated to be very low, suggesting that risk of bias was a serious concern. Besides that, there were some serious concerns identified regarding imprecision due to the small number of events that were included. Indirectness was not a concern, and publication bias was considered undetected because a potential conflict of interest in the studies included was not reported and the systematic review search strategy was wide, including grey literature. Inconsistency was not evaluated.

## DISCUSSION

To the best of our knowledge, this is the first systematic review that has evaluated children’s and parents’ preferences concerning physicians’ attire. Understanding these preferences/perceptions may be of great importance in facilitating a successful physician-patient relationship. Moreover, physicians’ attire can be interpreted as an indicator of professionalism, which could impact patient-doctor relationship. In general, most studies included in this systematic review have found that parents preferred physicians to be dressed in white coats.^5^ ^,^ ^6^ ^,^ ^11^ ^,^ ^22^ ^,^ ^25^ ^,^ ^26^ ^,^ ^27^ ^,^ ^29^ ^,^ ^31^ ^,^ ^32^ ^,^ ^33^

The studies included presented with diverse results. Previous research concluded that most mothers preferred children’s physicians to wear attire and that most caregivers preferred physicians to wear a white coat.^6^ The casually dressed pediatrician was the preferred attire and has not altered the parents’ perceived reliability on physicians.^23^ On the other hand, some authors found that parents prefer physicians wearing hospital scrubs and sneakers.^30^ Parents of patients with surgical emergencies were found to be more likely to prefer doctors wearing surgical scrubs.^22^ In another study, no preference for any particular style of physician attire was found.^24^

In addition, highlighting that previous studies have concluded that children may perceive physician attire differently from their parents is of utmost importance.^5^ ^,^ ^25^ ^,^ ^28^ Different variables and methodological aspects could have influenced in the results of those studies. As to preferences and the possible association with a child’s age, a likelihood for older children to prefer white coats and for younger ones to prefer informal attire was verified.^29^ Evaluating children’s preferences according to their developmental level is essential. Besides that, the research setting could also have influenced the findings. Children at the hospital have been found to have most frequently preferred the photo of physicians wearing a white coat. Of note, teenagers were found to prefer, in order, professional dress, semiformal, formal, and, finally, casual attire.^26^

A previous systematic review conducted in an adult population identified the influence of geographic location on attire preferences. Geographic location was found to influence perceptions of attire, perhaps demonstrating cultural, fashion, or ethnic expectations.^12^ Saudi national attire (thobe and shemagh) was one the most preferred attire indicated in Saudi Arabian research.^6^ Of note, one study concluded that the results did not differ significantly across age, gender, or number of hospitalizations.^28^ The severity of illness, type of health insurance, and age, race, and gender of guardians were found to not affect preferences.^22^

This systematic review has not confirmed the popular myth of the “white coat syndrome”. A previous systematic review carried out in adults reported that although patients often prefer formal physician attire (with or without white coat), this perception is complex and multifactorial.^12^

Despite this systematic review having had identified that many people across ages may prefer white coat attire, recommendations based on current legislation and biosecurity should be considered. A recent systematic review has compared the level of bacterial contamination between white coats and surgical scrubs. White coats and scrubs are commonly colonized with multidrug resistant organisms. According to the main findings, white coats are laundered much less frequently than surgical scrubs and, therefore, result in greater infection risk. Data regarding contamination based on fabric type are variable in findings. In addition, scrubs impregnated with antimicrobial substances can potentially reduce contamination. Laundering practices have a varying degree of efficacy in reducing contamination.^34^

Most of the selected studies in this review have demonstrated a low risk of bias. However, highlighting that the evaluation of physicians’ attire varied considerably between the studies is also crucial. In addition, there was marked substantial methodological variation across factors including the research settings (hospital waiting rooms, universities, medical clinics, and emergency services), age ranges of children, and geographic and cultural aspects of the samples evaluated. Multiple variables could have been associated with children’s and parents’ preferences concerning physicians’ attire.

Some limitations of this systematic review should be considered. All studies included were descriptive, conducted using convenience samples. Likely, future studies should systematically explore the effect of developmental levels of children on these preferences for physicians’ attires. Furthermore, other confounding factors such as gender, levels of anxiety, personality features, past medical experiences, and socioeconomic backgrounds should be considered for a better understanding of children’s and parents’ preferences. Cultural factors should also be explored in future studies. In addition, vote counting was applied to carry out data synthesis. Although that this method may be effective to assess the ranking of outcomes, it fails to account for the population size.^18^ Also, data analysis did not allow the proper access to the certainty of evidence with GRADE, since it was not possible to evaluate inconsistency.

Nowadays, due to the COVID-19 pandemic response, physician attire is increasingly mandated to include some or almost all available disposable PPI, including caps, goggles, face shields, N95 masks (sometimes with a surgical mask over them), gowns, and gloves.^35^ Future studies should address the parents’ and children’s perceptions and responses to COVID-19 standard personal protective equipment. The finds of this review suggest that healthcare systems should consider multiple factors, including context of care, when defining policies related to dress code.

In conclusion, parents and children preferred physicians to wear a white coat with very low certainty of evidence. Laws and regulations concerning wearing proper attire and protective clothing as well as equipment should be followed in order to protect both patients and healthcare providers from infectious diseases during the performance of medical care.
